# Celebrating *JSR*’s 30th anniversary: reminiscences of a Main Editor

**DOI:** 10.1107/S1600577524009482

**Published:** 2024-10-01

**Authors:** John R. Helliwell

**Affiliations:** a Department of Chemistry University of Manchester ManchesterM13 9PL United Kingdom

**Keywords:** *Journal of Synchrotron Radiation*, 30th anniversary

## Abstract

Reminiscences of one of the founding Main Editors of *JSR* on its 30th anniversary.

Thirty years ago, I was in the middle of my career at the Daresbury Laboratory Synchrotron Radiation Source, which ran from 1979 to 2008, mainly as a joint appointee but also with periods there full time. I was the founding chairman of the International Union of Crystallography (IUCr) Commission on Synchrotron Radiation (1990 to 1996). During that time, I was consulted by the IUCr’s Executive Committee about a proposal from Samar Hasnain for a *Journal of Synchrotron Radiation*. The alternative to such a new journal, it was explained to me, would be that *Journal of Applied Crystallography*, a successful IUCr journal for many years already, could be expanded to include a section on synchrotron radiation crystallography, diffraction and scattering. On reflecting on what advice I should give, I noted to myself that rather than choosing *Journal of Applied Crystallography* for my own synchrotron radiation instrumentation publications I had chosen non-IUCr journals, namely *Journal of Physics E* of the UK Institute of Physics Publishing and the journal *Nuclear Instruments and Methods* published by Elsevier. Additionally, in my new book, *Macromolecular Crystallography with Synchrotron Radiation*, published by Cambridge University Press in 1992, I had included EXAFS (extended X-ray absorption spectroscopy fine structure). Therefore, my advice to the IUCr Executive Committee was that the proposal of Samar Hasnain for a *Journal of Synchrotron Radiation* was a very good one, on academic grounds at least. There had to follow a business case to be made for the IUCr’s Finance Committee to evaluate. This was duly made and endorsed (see the companion article by Samar Hasnain to be published in a forthcoming issue). Within this there was a consultation of the wider synchrotron radiation community at conferences. I made some of these trips. I recall a largely, if not completely, positive response from audiences.

In moving ahead with *Journal of Synchrotron Radiation* (*JSR*) I was invited to be one of the Main Editors, along with Samar Hasnain and Hiromichi Kamitsubo. The IUCr was generous with paying for promotional leaflets for *JSR*. The calls for papers were only modestly successful, however, as measured by the several years when most of the bimonthly issues were barely above the minimum of 48 printed pages. A major burden of work on the editors in practice was that the synchrotron radiation community was, during that time, wedded to conference proceedings for both the Synchrotron Radiation Instrumentation (SRI) series and the EXAFS series of conferences. Fortunately, in my view, these conference proceedings became less and less required by the community in favour of a steady stream of articles in *JSR*.

In 1999 at the IUCr Congress in Glasgow I handed over my *JSR* Main Editor work to Denny Mills of the APS in Chicago, USA. This was because in 1996 I had become the Editor-in-Chief of *Acta Crystallographica* and Chairman of the IUCr’s Commission on Journals, a role I continued until the 2005 IUCr Congress.

The bimonthly issues of *JSR* have become very healthy in their number of articles per issue published. Within that trend I think a major and helpful step was the support of the individual synchrotron radiation facilities with their Facility Information pages included in *JSR* and linked with those the direct sponsorship of the journal by those facilities. Clearly the synchrotron radiation community is firmly supporting the journal, and no competitor has emerged over all these 30 years. Furthermore, *JSR*’s transition to being fully open access is a major contribution to the IUCr’s profile in open science which is a major modern trend today.

